# Long-term incidence of posterior capsular opacification in patients with non-infectious uveitis

**DOI:** 10.1038/s41598-022-08325-7

**Published:** 2022-03-11

**Authors:** Yuki Kitaguchi-Iwakiri, Koju Kamoi, Hiroshi Takase, Yusuke Okubo, Kyoko Ohno-Matsui

**Affiliations:** 1 Department of Ophthalmology & Visual Science, Graduate School of Medical and Dental Sciences, Tokyo Medical and Dental University (TMDU), 1-5-45 Yushima, Bunkyo-ku, Tokyo, 113-8519 Japan; 2 Division of Lifecourse Epidemiology, Department of Social Medicine, National Center for Child Health and Development, Tokyo, Japan; 3 Department of Epidemiology, UCLA Fielding School of Public Health, Los Angeles, CA USA

**Keywords:** Lens diseases, Uveal diseases

## Abstract

Little is known about the long-term incidence of posterior capsule opacification (PCO) after cataract surgery in patients with uveitis. This retrospective study included 211 eyes of 146 patients with non-infectious uveitis who underwent cataract surgery and implantation of an Acrysof SN60WF (Surface: plasma-treated, Optic and Haptic: hydrophobic acrylic), iSert XY-1 (Surface: UV-ozone-treated, Optic and Haptic: hydrophobic acrylic), or iSert 251/255 (Surface: UV-ozone-treated, Optics: hydrophobic acrylic, Haptic: polymethyl methacrylate). The cumulative incidences of PCO and subsequent yttrium–aluminum-garnet (Nd:YAG) capsulotomy over the 5-year follow-up were analyzed, and patients who were implanted with different intraocular lenses (IOLs) were compared. Mixed-effects Cox proportional hazard models showed that, compared with the Acrysof group, the iSert XY-1 group had higher risks of PCO (adjusted HR, 7.26; 95% CI, 1.82–28.8) and Nd:YAG capsulotomy (adjusted HR, 6.50; 95% CI, 1.55–27.2). Similar results were obtained when the Acrysof group was compared with the iSert 251/255 group for PCO (adjusted HR, 8.22; 95% CI, 2.35–28.7) and Nd:YAG capsulotomy (adjusted HR, 8.26; 1.90–36.0). These data suggest that a plasma-treated surface, hydrophobic acrylic optic and hydrophobic acrylic haptic, of the IOL could enhance biocompatibility even under inflammatory conditions, thus suppressing PCO development.

## Introduction

Cataract formation is one of the major complications of intraocular inflammation (i.e., uveitis)^1^. Although implantation of an intraocular lens (IOL) is associated with a risk of exacerbated inflammation, most cataract surgeries for uveitis nevertheless involve IOL implantation. Indeed, previous studies have reported that IOLs are effective even in patients with ocular inflammation^2–5^.

The incidence of posterior capsule opacification (PCO) and subsequent neodymium: yttrium–aluminum-garnet (Nd:YAG) capsulotomy following cataract surgery is thought to depend on the biomaterials used for IOLs and their optic and haptic design characteristics^6,7^. PCO is thought to be caused by the proliferation and subsequent migration of residual lens epithelial cells (LECs) from the peripheral posterior capsular bag into the space below the IOL optic^8^. Inflammation is believed to accelerate LEC proliferation and migration, since cytokines and chemokines can more easily flow into the eye and stimulate LECs following the breakdown of blood-ocular barriers^9^.

Studies of non-inflamed normal eyes have identified several important factors related to PCO development, such as surgical technique, age, sex, and diabetes mellitus. In terms of biomaterials, hydrophobic IOLs are associated with a lower incidence of PCO and subsequent Nd:YAG capsulotomy than hydrophilic IOLs^10^. With regard to design parameters, the incidence of PCO after implantation of IOLs with sharp edges is reportedly lower than that of implantation of IOLs with duller edges^7^. However, little is known about the incidence of PCO and subsequent Nd:YAG capsulotomy in patients with uveitis over the long term. Therefore, which features of IOL are most appropriate for patients with underlying uveitis remain unclear.

In this study, the incidences of PCO and subsequent Nd:YAG capsulotomy were analyzed and compared in patients with non-infectious uveitis who were implanted with IOLs having different features over a long follow-up period.

## Results

The study involved a total of 211 eyes of 146 patients with non-infectious uveitis (124 eyes of 90 patients with the Acrysof SN60WF (Alcon Surgical Inc., Fort Worth, TX, USA), 50 eyes of 33 patients with the iSert XY-1 IOL (HOYA Co. Ltd., Tokyo, Japan), and 37 eyes of 23 patients with the iSert 251/255 IOL (HOYA Co. Ltd.). The average age of the patients was 60.7 years (SD, 14.0 years). Overall, 36.3% of patients had sarcoidosis, and 18.6% had diabetes mellitus. Details of the baseline characteristics of the Acrysof SN60WF, iSert XY-1, and iSert 251/255 groups are summarized in Table 1.

**Table 1:** Baseline characteristics stratified by treatment group.

Treatment group	Acrysof (SN60WF)	iSert XY-1	iSert 251/255	Total
Individuals, N	90	33	23	146
**Eyes, N**	124	50	37	211
Right	61	25	15	101
Left	63	25	22	110
Age, mean (SD)	60.0 (13.7)	62.9 (14.3)	59.7 (14.6)	60.7 (14.0)
Age, range	20–82	36–81	28–85	20–85
**Sex, N (%)**				
Male	38 (30.6%)	15 (30%)	13 (35.1%)	66 (30.7%)
Female	86 (69.4%)	35 (70%)	24 (64.9%)	145 (69.3%)
**Comorbidity, N (%)**				
Diabetes mellitus	22 (17.7%)	10 (20%)	8 (21.6%)	40 (18.6%)
**Entity N (%)**				
Idiopathic uveitis	71 (57.2%)	22 (44.0%)	12 (32.4%)	105 (49.8%)
Sarcoidosis	43 (34.7%)	19 (38%)	16 (43.2%)	78 (36.3%)
Vogt-Koyanagi-Harada disease	8 (6.5%)	0 (0%)	2 (5.4%)	10 (4.7%)
LogMAR, mean (SD)	1.3 (1.1)	1.6 (1.2)	1.1 (1.1)	1.3 (1.1)
Refraction, mean (SD)	− 2.0 (2.9)	− 2.3 (2.9)	− 3.3 (3.2)	− 2.3 (3.0)

*SD* standard deviation, *LogMAR* log of the minimum angle of resolution.

**Figure 1:**
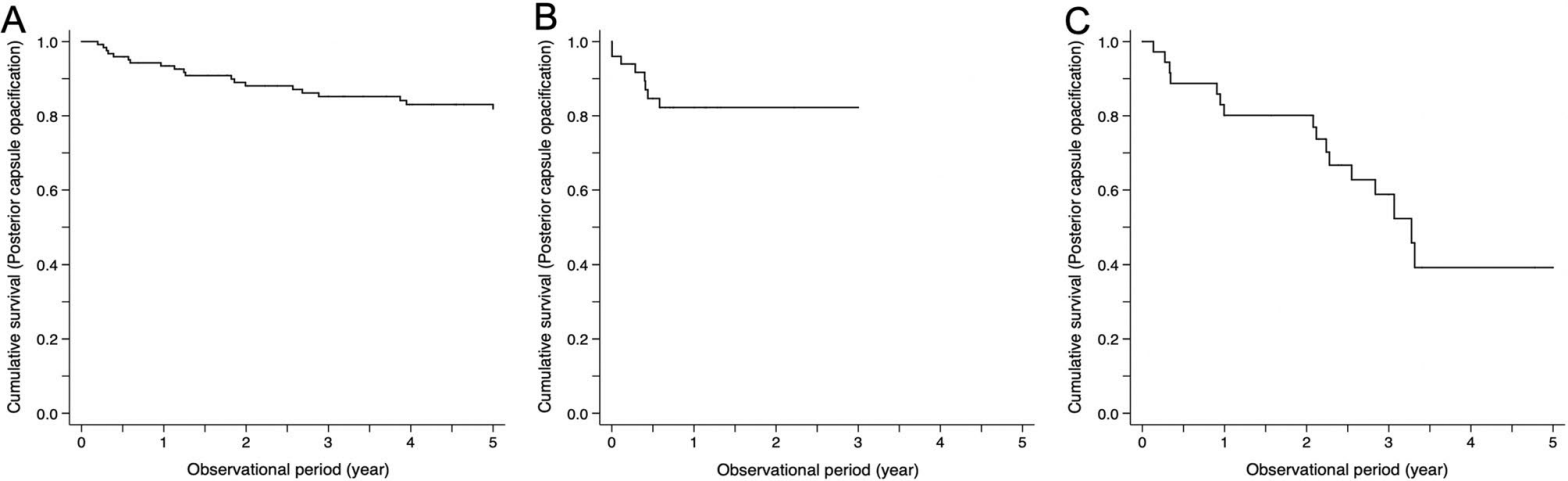
Cumulative survival curves of posterior capsule opacification (PCO). (**A**) Acrysof group. (**B**) iSert XY-1 group. (**C**) iSert 251/255 group. A difference is observed at the initial time point, with the Acrysof group exhibiting a significantly lower incidence of PCO than the iSert XY-1 and iSert 251/255 groups over the 5 years (log-rank test, p < 0.001).

In all enrolled patients, the cumulative incidences of PCO and Nd:YAG capsulotomy were examined from 3 months to 5 years. The cumulative incidences of PCO were 16.2% in the Acrysof SN60WF group, 28.6% in the iSert XY-1 group, and 48.3% in the iSert 251/255 group at 3 years (Supplemental Table 2). Kaplan–Meier survival curves for different lens groups are presented in Fig. 1; stratified data stratified by age, diabetes mellitus, and sarcoidosis are in Supplemental Fig. 1. In the Acrysof SN60WF group, patients with sarcoidosis were less likely to have PCO and Nd:YAG during follow-up (Supplemental Figs. 1C, 2C).

**Figure 2:**
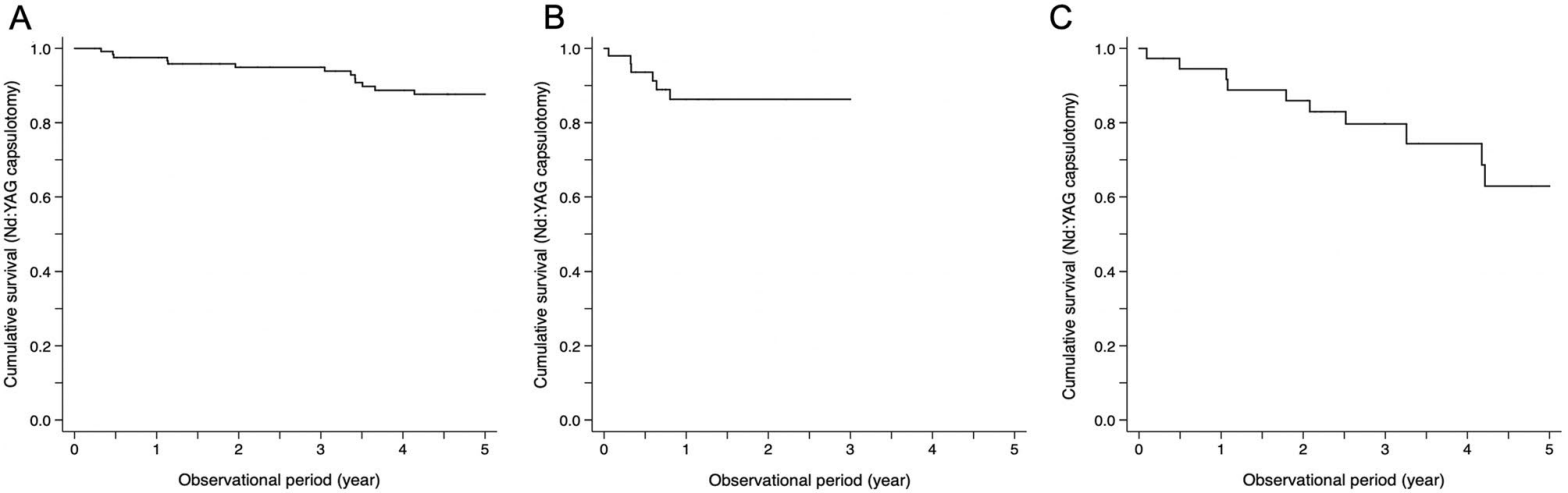
Cumulative survival curves of Nd:YAG capsulotomy. (**A**) Acrysof group. (**B**) iSert XY-1 group. (**C**) iSert 251/255 group. A difference is observed at the initial time point. The incidence of Nd:YAG capsulotomy over the 5-year follow-up is significantly lower in the Acrysof group than in the iSert group (log-rank test, p < 0.001).

The cumulative incidences of Nd:YAG capsulotomy were 6.7% in the Acrysof SN60WF group, 21.4% in the iSert XY-1 group, and 23.3% in the iSert 251/255 group at 3 years (Supplemental Table 2). Kaplan–Meier survival curves for different lenses are presented in Fig. 2; stratified data by age, diabetes mellitus, and sarcoidosis are in Supplemental Fig. 2. The cumulative incidence of Nd:YAG capsulotomy at 3-year follow-up was significantly higher in the iSert XY-1 (adjusted risk ratio, 4.42; 95% CI, 1.18–16.5) and iSert 251/255 groups (adjusted risk ratio, 4.56; 95% CI, 1.01–20.5) than in the Acrysof SN60WF group (Table 2).

**Table 2:** Cumulative incidence ratio (risk ratio) for posterior capsule opacification and Nd:YAG capsulotomy, and mean differences in logMAR levels over 36-month follow-up between the Acrysof (SN60WF, reference) and iSert groups (XY-1 or 251/255, index).

Comparison	iSert XY-1 vs. Acrysof (SN60WF)		iSert 251/255 vs. Acrysof (SN60WF)	
Analyses	Crude	Adjusted*^1^	Crude	Adjusted*^1^
Effect estimate	Risk ratio (95% CI)		Risk ratio (95% CI)	
**Posterior capsule opacification**				
36 months	1.76 (0.93, 3.35)	0.99 (0.43, 2.28)	2.98 (1.81, 4.92)	2.39 (1.50, 3.79)
**Nd:YAG capsulotomy**				
36 months	3.21 (1.05, 9.80)	4.42 (1.18, 16.5)	3.50 (1.20, 10.21)	4.56 (1.01, 20.5)
**LogMAR, Mean Difference (95% CI)**				
3 months	0.00 (− 0.26, 0.26)	0.00 (− 0.26, 0.25)	− 0.14 (− 0.43, 0.15)	− 0.14 (− 0.43, 0.14)
6 months	− 0.09 (− 0.36, 0.17)	− 0.07 (− 0.33, 0.19)	− 0.15 (− 0.43, 0.13)	− 0.15 (− 0.43, 0.12)
12 months	− 0.05 (− 0.33, 0.23)	− 0.04 (− 0.31, 0.24)	− 0.09 (− 0.38, 0.21)	− 0.10 (− 0.39, 0.19)
36 months	− 0.09 (− 0.42, 0.24)	− 0.07 (− 0.40, 0.26)	− 0.10 (− 0.42, 0.23)	− 0.12 (− 0.44, 0.20)

For the adjusted model, age, sex, diabetes mellitus, and sarcoidosis were included as covariates (*1).

*SD* standard deviation, *CI* confidence interval, *LogMAR* log of the minimum angle of resolution.

The hazards of PCO and Nd:YAG capsulotomy were compared among the Acrysof SN60WF, iSert XY-1, and iSert 251/255 groups using mixed-effects Cox proportional hazards models (Table 3). The hazards of PCO were higher in the iSert XY-1 and iSert 251/255 groups than in the Acrysof SN60WF group. Correspondingly, the hazard of Nd:YAG capsulotomy was higher in the iSert XY-1 and 251/255 groups than in the Acrysof group. When the same analyses were performed using data over the first 3 years, the directions of effect estimates were identical to the primary analyses (Supplemental Table 3).

**Table 3:** Hazard ratios of posterior capsule opacification and Nd:YAG capsulotomy, comparing the iSert XY-1 and iSert 251/255 as the index group with the Acrysof (SN60WF) as the reference group using data over the 5-year follow-up period.

Treatment groups		Acrysof (SN60WF)	iSert XY-1	iSert 251/255
**Posterior capsule opacification**				
Crude model	Hazard ratio (95% CI)	Ref	4.54 (1.20, 17.2)	4.88 (1.52, 15.6)
Adjusted model*	Hazard ratio (95% CI)	Ref	7.26 (1.82, 28.8)	8.22 (2.35, 28.7)
**Nd:YAG capsulotomy**				
Crude model	Hazard ratio (95% CI)	Ref	5.89 (1.28, 27.0)	7.32 (1.58, 33.8)
Adjusted model*	Hazard ratio (95% CI)	Ref	6.50 (1.55, 27.2)	8.26 (1.90, 36.0)

Variables included as covariates in the adjusted model were age, sex, diabetes mellitus, and sarcoidosis. The Acrysof IOL (SN60WF) was defined as the reference group and the iSert (XY1 or 251/255) group as the index group.

*CI* confidence interval, *Ref.* reference.

**Figure 3:**
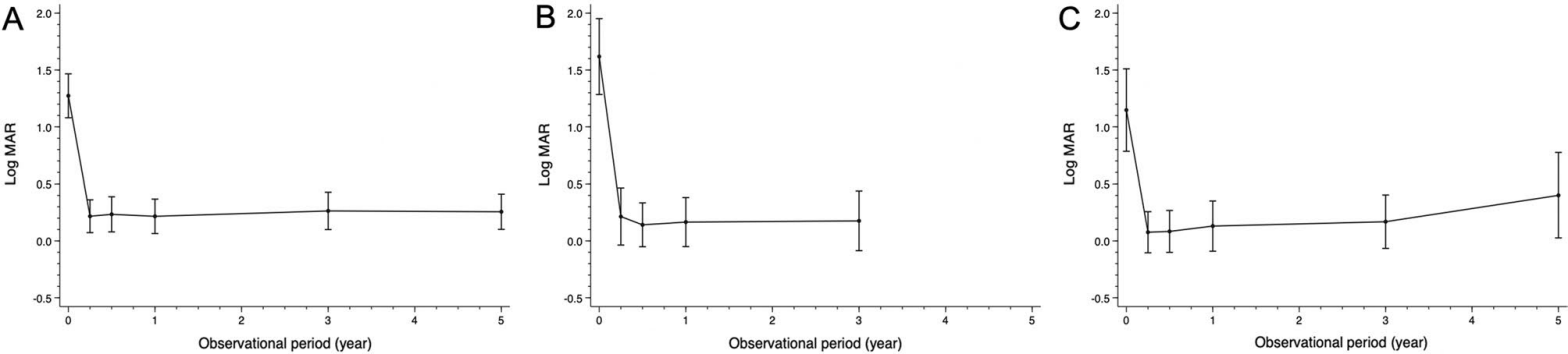
Changes in Visual acuity (VA). (**A**) Acrysof group. (**B**) iSert XY-1 group. (**C**) iSert 251/255 group. A significant improvement in VA (logMAR) is observed after cataract surgery in all groups. However, there is no significant difference in VA among groups at each time point, but slightly better VA is observed in the Acrysof group at 5 years.

Similar improvements in VA (logMAR; Fig. 3, Table 2) after cataract surgery were observed in the Acrysof SN60WF group, iSert XY-1 group, and iSert 251/255 group. The trends in logMAR levels were similar among the Acrysof SN60WF, iSert XY-1, and iSert 251/255 groups at any time point over the follow-up period.

## Discussion

Intraocular inflammation may affect the biocompatibility of IOLs and subsequently play a role in the development of PCO after cataract surgery. For example, a previous study at a single institution in the US showed that patients with uveitis had a higher incidence of PCO following cataract surgery involving the implantation of an IOL than patients without uveitis^11^. Furthermore, a previous study and the present findings may suggest that different types of IOLs have different risks of PCO after cataract surgery. For instance, a study reported that the risk of PCO was approximately 13% at 3-year follow-up after surgery in patients receiving Acrysof SN60WF IOLs in non-inflamed eyes^12^. Similarly, in the present study, the risk of PCO at 3 years was 16.2% even in the presence of intraocular inflammation (Fig. 1, Supplemental Table 2). In comparison, a previous study reported that the risk of PCO at 1 year in patients receiving the iSert XY-1 in non-inflamed eyes was 8.5%^13^, whereas the present study showed that the risk of PCO in the iSert group at 1 year in inflamed eyes was 28.6% (Fig. 1, Supplemental Table 2). This sharp contrast suggests that PCO is more likely to develop in patients implanted with iSert XY-1 and iSert 251/255 in an inflamed eye than in those with Acrysof SN60WF. Therefore, the Acrysof SN60WF may contain materials, surface treatments, or designs (optic edge/haptic loop) that inhibit the development of PCO in inflamed eyes, although which elements contribute to the incidence of PCO in patients with uveitis are unclear^14^.

Regarding the optical part of IOL materials, a previous study found that uveitis patients implanted with acrylic IOLs had a lower incidence of PCO than patients with IOLs made of other materials (i.e., silicone or PMMA)^15^. The lower incidence of PCO in the hydrophobic acrylic IOLs could be explained by several factors. First, the hydrophobic acrylic IOLs exhibit greater bioadhesion of fibronectin, which promotes adhesion to the capsular bag even in patients with uveitis^15^. Second, IOL adhesion to LECs is directly correlated with a decrease in the capsule contraction rate. Indeed, it was reported that Acrysof SN60WF IOLs adhered to LECs at a higher rate than lenses made of other materials^16^. Therefore, either Acrysof or iSert IOLs are currently used to lower the incidence of PCO, even in patients with uveitis. Surprisingly, different risks of PCO were observed across different types of IOLs, although the Acrysof and iSert IOLs were all fabricated of hydrophobic acrylic material (Supplemental Table 1). The results suggest that the differences in PCO incidence between these IOLs were dependent on characteristics other than hydrophobic acrylic material.

With regard to surface treatments of IOLs, the Acrysof (SN60WF) IOL is plasma-treated, whereas the iSert IOLs are UV/ozone-treated on the backside of the surface (Supplemental Table 1). A previous investigation showed that both plasma and UV/ozone treatment of the IOL surface effectively prevented PCO after cataract surgery^17,18^. Although the differences in surface treatment of the lenses (plasma or UV/ozone) are, in general, unlikely to affect PCO incidence in uninflamed eyes, the present findings (Acrysof vs. iSert 251/255) suggest that the different surface treatments may have contributed to the development of PCO to some extent in inflamed eyes.

As for the haptic elements, the Acrysof, iSert XY-1, and iSert 251/255 consisted of C-loop haptics (Supplemental Table 1). Therefore, it appears that the haptics designs were unlikely to affect the risks of PCO and Nd:YAG capsulotomy in the present study. On the other hand, with regard to haptics materials, Acrysof, iSert XY-1, and iSert 251/255 had different haptic edges (Acrysof and XY-1: hydrophobic acrylic haptic edge, 251/255: PMMA haptic edge) in the present study, which may have contributed to the differences in the risks of PCO and Nd:YAG capsulotomy over the 3–5 years (Figs. 1 and 2, Tables 2 and 3, and Supplemental Tables 2 and 3).

Taken together, surface treatment of the IOL and the haptics material may have affected the incidences of PCO and subsequent Nd:YAG capsulotomy; the plasma-treated IOL with a hydrophobic acrylic haptic edge had a better outcome than the ozone-treated IOL with a PMMA haptic edge. It is possible that LECs tended to proliferate on the anterior side over the IOL under inflammatory conditions due to the presence of cytokines and chemokines in the aqueous humor. These LECs could have then more easily migrated to the posterior capsule because of the UV/ozone-treated surface and the PMMA material of the haptics, thereby contributing to the higher incidence of PCO (Figs. 1, 2, Tables 2, 3, and Supplemental Tables 2, 3).

The present study had several limitations. Although the cataract surgeries were performed by two experienced surgeons who specialized in uveitis and used the same instruments and phaco-machines, and performed the same procedure indicated in the Methods, surgery could have had an effect on the outcome of PCO. Whether to perform Nd:YAG capsulotomy requires consensus between patients and ophthalmologists regarding the indications, which could have caused a slight delay when patients were hesitant to undergo Nd:YAG capsulotomy, although effects from any such delays were minimal in long-term follow-up. Although many PCO evaluation systems are currently available, slit-lamp grading and fundus visualization were selected because the present study focused on enrolling a number of patients and following them over a long period (5 years). Further evaluations using other systems such as density mapping may be appropriate in future studies.

Due to the nature of the present study design, the findings could have been affected by certain inherent biases: confounding due to unmeasured variables and selection bias due to right censoring. The generalizability of the findings to the usual healthcare setting is unclear because this study was conducted at a single institution. However, the enrolled patients reflected the epidemiology of uveitis in Japan. The most frequent cause of uveitis in Japan was sarcoidosis (10.6%), whereas JIA was seen in 0.5%, according to a recent nationwide survey^19^. This survey showed that young patients with uveitis (< 20 years old) accounted for 5.6%, whereas old patients with uveitis (> 60 years old) accounted for 46.3%. The characteristics of uveitis in Japan, such as fewer young patients and anterior uveitis, might have affected the results, which could be a potential bias and should be addressed in the future^20^. In addition, identifying different clinical courses in patients with different baseline characteristics is important for precision medicine. To ascertain the clinical courses (PCO development and Nd:YAG capsulotomy) by age, diabetes mellitus, and sarcoidosis with each type of IOL, stratified analyses were performed. It was found that patients without sarcoidosis had lower cumulative survivals for PCO and Nd:YAG capsulotomy. Although elderly persons (age > 65 years) and the presence of diabetes mellitus appeared to have different survival curves, there were no significant differences, indicating the need for larger sample sizes. Due to these limitations, well-designed, prospective studies are necessary to determine the differences in the risks of PCO and Nd:YAG capsulotomy among the different hydrophobic acrylic IOLs.

In conclusion, this study demonstrated that the Acrysof SN60WF IOLs were associated with significantly lower incidences of PCO and subsequent Nd:YAG capsulotomy compared to the iSert XY-1 and iSert 255/251 IOLs in patients with uveitis. A hydrophobic acrylic haptics edge and plasma-treated surface of the IOL could enhance biocompatibility even under inflammatory conditions, thus suppressing PCO development. In selecting an IOL for patients with uveitis, the fabrication material of the haptic edge and any surface treatments applied should be considered to minimize the risk of developing PCO, since the incidence of PCO differed significantly during long-term follow-up.

## Methods

The study protocol conformed to the tenets of the Declaration of Helsinki and was approved by the Ethics Committee of Tokyo Medical and Dental University. The need for informed consent was waived by the Ethics Committee of Tokyo Medical and Dental University due to the retrospective nature of this study. The study included IOLs implanted after cataract surgery in patients with non-infectious uveitis at the Tokyo Medical and Dental University Hospital between January 2012 and August 2017. Each patient had given informed consent to phacoemulsification and IOL implantation. As for IOL type, Acrysof SN60WF was used in the beginning of the study period and iSert 251/255 followed by iSert XY-1 was used during the later period after a hospital decision based on cost considerations.

Exclusion criteria for the study included a history of previous corneal lesions such as corneal opacity, lens abnormalities such as pseudoexfoliation syndrome, retinal abnormalities such as macular degeneration, ocular trauma, and previous ocular surgical procedures or surgical complications. A primary posterior capsulotomy or anterior vitrectomy in combination with cataract surgery was also excluded.

Two experienced surgeons specializing in uveitis (KK and HT) performed all surgeries using the same surgical procedure and topical anesthesia. The surgical procedures were standardized^21–23^. A 2.5-mm, clear corneal/scleral incision was created at the 12 o’clock position. Sodium hyaluronate (1%) was used as the ophthalmic visco surgical device, and a balanced saline solution was used for hydrodissection. A well-centered continuous curvilinear capsulorhexis with a diameter of approximately 5–6 mm was then created. After nucleus removal via phacoemulsification, the capsular bag was filled with viscoelastic fluid, and the IOL was implanted in the bag using an injector system. Postoperatively, topical dexamethasone applied 4–6 times daily was prescribed, with tapering depending on improvement of ocular inflammation.

The incidences of PCO and subsequent Nd:YAG capsulotomy were examined retrospectively for the IOLs most commonly used in cataract surgery in Japan, the Acrysof SN60WF (Alcon Surgical Inc., Fort Worth, TX, USA), and the iSert XY-1 and iSert 251/255 (HOYA Co. Ltd., Tokyo, Japan).

As for the features of the IOLs, the Acrysof SN60WF is constructed of plasma-treated, sharp-edged, hydrophobic, acrylic optic material and hydrophobic, acrylic haptic material with a C-loop. The iSert XY-1 is constructed of UV-ozone-treated hydrophobic, acrylic, sharp-edged optic material and hydrophobic, acrylic haptic material with a C-loop. The iSert 250/251 has UV-ozone-treated, hydrophobic, acrylic, sharp-edged optic material and polymethyl methacrylate (PMMA) haptic material with a C-loop (Supplemental Table 1).

PCO was identified from the medical records based on slit-lamp grading and fundus visualization (Grade 1)^24,25^. To confirm PCO incidence more objectively, the incidence of Nd:YAG capsulotomy subsequent to IOL implantation was also evaluated^26^. Indications for YAG capsulotomy included significant biomicroscopic evidence of PCO (Grade 1–3) along with a subjective visual complaint, including decreased visual acuity, severe glare, or both^11,25^. The incidence of PCO with subsequent Nd:YAG capsulotomy and visual acuity (VA) were evaluated in each patient at 3 months, 6 months, 1 year, 3 years, and 5 years. VA was evaluated at each follow-up point as supportive data^26,27^.

All data were analyzed using Stata/MP software version 16.1 (StataCorp LP, TX, USA) with the following five steps. First, descriptive statistics were summarized as means (standard deviations [SDs]) or proportions, as appropriate. Second, the cumulative incidence (risk) of PCO and Nd:YAG capsulotomy and the mean logarithm of the minimum angle of resolution (logMAR) were determined across different lens groups. Cumulative survivals for PCO and Nd:YAG were also presented across different lens groups using Kaplan–Meier curves: the data were stratified by age (≤ 65 years and > 65 years)^28,29^ and the comorbidities of diabetes mellitus and sarcoidosis (Supplemental Figs. 1 and 2). Third, risks of PCO and Nd:YAG capsulotomy over different follow-up periods (36 months) were compared among the Acrysof SN60WF, iSert XY1, and iSert 251/255 groups. Generalized linear models were constructed under log-link functions and binomial distributions to estimate the risk ratios of PCO and Nd:YAG capsulotomy. Age, sex, diabetes mellitus, and sarcoidosis were included as potential confounders in the models. Bootstrapping was used to calculate 95% confidence intervals (CIs). Fourth, mixed-effects Cox proportional hazard models with robust variance estimates, unstructured covariance, and random effects for clustering the eyes in individuals were developed to estimate the hazard ratios (HRs) of the incidences of PCO and Nd:YAG capsulotomy among the Acrysof SN60WF, iSert XY1, and iSert 251/255 groups^30^. The crude and adjusted HRs are reported using the data for 5 years as the primary analyses and the data during the first 3 years as the sensitivity analyses; for the adjusted analyses, covariates (age, sex, comorbidities of diabetes mellitus and sarcoidosis) were included in the crude models. Fifth, the mean logMAR (SD) was compared across the Acrysof SN60WF, iSert XY1, and iSert 251/255 groups at 3, 6, 12, and 36 months of follow-up using multivariable linear regression models.

## Supplementary Information

Supplementary Information.
