# Supplementary material for: Long-term incidence of posterior capsular opacification in patients with non-infectious uveitis
Source: Sci Rep. 2022 Mar 11;12:4296. doi: 10.1038/s41598-022-08325-7 (PMC8917155; doi:10.1038/s41598-022-08325-7)
Supplement: Supplementary file 1 — Supplementary Information. [file 41598_2022_8325_MOESM1_ESM.docx]

**Supplementary Material**

**Supplemental Tables**

**Supplementary Table 1.** Features of intraocular lenses.

| **Features** | | **Acrysof (SN60WF)** | | | **iSert XY-1** | | | **iSert 251/255** | | |  |
| --- | --- | --- | --- | --- | --- | --- | --- | --- | --- | --- | --- |
| **Surface** | | | |  | | |  | | |  |  |
| Treatment  **Optic**  Material  Edge |  | | Plasma-treated  Hydrophobic acrylic  Sharp | | | UV-ozone-treated  Hydrophobic acrylic  Sharp | | | UV-ozone-treated  Hydrophobic acrylic  Sharp | | |
| **Haptic**  Material  Loop |  | | Hydrophobic acrylic  C-loop | | | Hydrophobic acrylic  C-loop | | | Polymethyl methacrylate  C-loop | | |

**Supplementary Table 2**. Cumulative incidences (posterior capsule opacification, Nd:YAG capsulotomy) and mean logMAR levels over the 60 months of follow-up between the Acrysof (SN60WF; reference) and iSert groups (XY1 or 251/255; index).

| **Treatment** | **Acrysof (SN60WF)** | **iSert XY-1** | **iSert 251/255** |
| --- | --- | --- | --- |

| **Eyes, N** | 124 | 50 | 37 |
| --- | --- | --- | --- |

| **Posterior capsule opacification, cases/N (Cumulative incidence (%))** |
| --- |

| 3 months | 4/124 (3.2%) | 3/50 (6.0%) | 3/37 (8.1%) |
| --- | --- | --- | --- |
| 6 months | 6/122 (4.9%) | 7/44 (15.9%) | 4/36 (11.1%) |
| 12 months | 9/120 (7.5%) | 8/39 (20.5%) | 7/36 (19.4%) |
| 36 months | 17/105 (16.2%) | 8/28 (28.6%) | 14/29 (48.3%) |

| 60 months | 20/90 (22.2%) | N/A^*1^ | 16/22 (72.7%)^*1^ |
| --- | --- | --- | --- |
| **Nd:YAG capsulotomy, cases/N (Cumulative incidence (%))** | | | |

| 3 months | 0/124 (0%) | 1/50 (2.0%) | 1/37 (2.7%) |
| --- | --- | --- | --- |
| 6 months | 2/122 (1.6%) | 3/44 (6.8%) | 1/36 (2.8%) |
| 12 months | 4/120 (3.3%) | 6/39 (15.4%) | 1/35 (2.9%) |
| 36 months | 7/105 (6.7%) | 6/28 (21.4%) | 7/30 (23.3%) |

| 60 months | 13/88 (14.8%) | N/A^*1^ | 10/20 (50.0%)^*1^ |
| --- | --- | --- | --- |
| **LogMAR, mean (SD)** | |  |  |

| 3 months | 0.22 (0.81) | 0.21 (0.88) | 0.08 (0.54) |
| --- | --- | --- | --- |
| 6 months | 0.23 (0.84) | 0.14 (0.63) | 0.08 (0.54) |
| 12 months | 0.22 (0.83) | 0.17 (0.66) | 0.13 (0.64) |
| 36 months | 0.26 (0.84) | 0.18 (0.66) | 0.17 (0.62) |

| 60 months | 0.26 (0.73) | N/A^*1^ | 0.40 (0.75) ^*1^ |
| --- | --- | --- | --- |

**Abbreviations:** SD, standard deviation; CI, confidence interval; LogMAR, log of the minimum angle of resolution

**Note:** Outcomes were not analyzed at 60 months because of the right censoring among all individuals who received the iSert XY-1 (*1).

**Supplemental Table 3.** Hazard ratios of posterior capsule opacification and Nd:YAG capsulotomy, comparing the iSert XY-1 and iSert 251/255 as the index group with the Acrysof (SN60WF) as the reference group using data during the first 3 years as a sensitivity analysis.

| **Treatment groups** | **Acrysof (SN60WF)** | **iSert XY-1** | | **iSert 251/255** |
| --- | --- | --- | --- | --- |
| **Posterior capsule opacification** | | |  |  |

| Crude model | Hazard ratio (95%CI) | Ref. | 3.37  (0.98, 11.56) | 3.55  (1.15, 10.9) |
| --- | --- | --- | --- | --- |
| Adjusted model* | Hazard ratio (95%CI) | Ref. | 5.28  (1.46, 19.1) | 5.86  (1.76, 19.5) |

| **Nd:YAG capsulotomy** |  |  |
| --- | --- | --- |

| Crude model | Hazard ratio (95%CI) | Ref. | 4.47  (0.89, 22.42) | 6.74  (1.36, 33.3) |
| --- | --- | --- | --- | --- |
| Adjusted model* | Hazard ratio (95%CI) | Ref. | 4.20  (0.96, 18.44) | 8.07  (1.65, 39.3) |

**Abbreviations:** CI, confidence interval; Ref., reference

**Note:** Variables included in the adjusted model were age, sex, entity, and comorbidity as covariates. The Acrysof IOL (SN60WF) was defined as the reference group and the iSert (XY1 or 251/255) group as the index group.

**Supplemental Figure 1A.** Kaplan-Meier survival curves for posterior capsule opacification (PCO) across different lens groups (Acrysof SN60WF, iSert XY-1, and iSert 251/255) stratified by age group (> 65 years vs. ≤ 65 years).

| **Acrysof SN60WF group** | **iSert XY-1** |
| --- | --- |
|  |  |
| **iSert 251/255** |  |
|  |  |

Note: Log-rank tests were used to compare the different age and comorbidity groups.

**Supplemental Figure 1B.** Kaplan-Meier survival curves for posterior capsule opacification (PCO) across different lens groups (Acrysof SN60WF, iSert XY-1, and iSert 251/255) stratified by diabetes mellitus.

| **Acrysof SN60WF group** | **iSert XY-1** |
| --- | --- |
|  |  |
| **iSert 251/255** |  |
|  |  |

Note: Log-rank tests were used to compare the different age and comorbidity groups.

**Supplemental Figure 1C.** Kaplan-Meier survival curves for posterior capsule opacification (PCO) across different lens groups (Acrysof SN60WF, iSert XY-1, and iSert 251/255) stratified by sarcoidosis.

| **Acrysof SN60WF group** | **iSert XY-1** |
| --- | --- |
|  |  |
| **iSert 251/255** |  |
|  |  |

Note: Log-rank tests were used to compare the different age and comorbidity groups.

**Supplemental Figure 2A.** Kaplan-Meier survival curves for neodymium:yttrium-aluminum-garnet (Nd:YAG) capsulotomy following cataract surgery across different lens groups (Acrysof SN60WF, iSert XY-1, and iSert 251/255) stratified by age group (> 65 years vs. ≤ 65 years).

| **Acrysof SN60WF group** | **iSert XY-1** |
| --- | --- |
|  |  |
| **iSert 251/255** |  |
|  |  |

Note: Log-rank tests were used to compare the different age and comorbidity groups.

**Supplemental Figure 2B.** Kaplan-Meier survival curves for neodymium:yttrium-aluminum-garnet (Nd:YAG) capsulotomy following cataract surgery across different lens groups (Acrysof SN60WF, iSert XY-1, and iSert 251/255) stratified by diabetes mellitus.

| **Acrysof SN60WF group** | **iSert XY-1** |
| --- | --- |
|  |  |
| **iSert 251/255** |  |
|  |  |

Note: Log-rank tests were used to compare the different age and comorbidity groups.

**Supplemental Figure 2C.** Kaplan-Meier survival curves for neodymium:yttrium-aluminum-garnet (Nd:YAG) capsulotomy following cataract surgery across different lens groups (Acrysof SN60WF, iSert XY-1, and iSert 251/255) stratified by sarcoidosis.

| **Acrysof SN60WF group** | **iSert XY-1** |
| --- | --- |
|  |  |
| **iSert 251/255** |  |
|  |  |

Note: Log-rank tests were used to compare the different age and comorbidity groups.
